# Comparison of Intracranial and Extracranial Carotid Artery Calcifications between Obstructive Sleep Apnea Patients and Healthy Individuals: A Combined Cone-Beam Computed Tomography and Polysomnographic Study

**DOI:** 10.1155/2022/1625779

**Published:** 2022-07-09

**Authors:** Mujgan Firincioglulari, Secil Aksoy, Kaan Orhan, Finn Rasmussen

**Affiliations:** ^1^Cyprus International University, Faculty of Dentistry, Department of Dentomaxillofacial Radiology, Nicosia, Cyprus; ^2^Near East University, Faculty of Dentistry, Department of Dentomaxillofacial Radiology, Nicosia, Cyprus; ^3^Ankara University, Faculty of Dentistry, Department of Dentomaxillofacial Radiology, Ankara, Turkey; ^4^Ankara University Medical Design Application and Research Center (MEDITAM), Ankara, Turkey; ^5^Internal Medicine Department Lunge Section, SVS Esbjerg, Esbjerg, Denmark; ^6^Life Lung Health Center Nicosia, Strovolos, Cyprus

## Abstract

**Purpose:**

This study aimed to compare the presence and grades of intra- and extracranial carotid artery calcifications between obstructive sleep apnea (OSA) and non-OSA patients.

**Methods:**

CBCT records of 190 patients (95 OSA patients and 95 non-OSA patients) were retrospectively collected and analyzed. Patient demographic data, including age and gender for both study groups and body mass index (BMI), and apnea-hypopnea index (AHI) for OSA patients were recorded. The presence of intra- and extracranial carotid artery calcifications and the number of calcifications were noted according to the grading scale.

**Results:**

There was a significant difference in carotid artery calcifications between OSA patients and healthy individuals. A total of 56.8% of the OSA patients showed at least one carotid artery calcification, whereas 13.8% of healthy individuals showed at least one carotid artery calcification (*p* < 0.05). For intracranial calcifications, OSA patients showed a significantly higher prevalence than healthy individuals (*p* < 0.05). The results showed that as the apnea-hypopnea index increases in OSA patients, the incidence of carotid artery calcification increases simultaneously. AHI > 30 patients showed the highest percentage of calcifications.

**Conclusion:**

In conclusion, OSA patients showed a higher prevalence of calcified carotid artery calcifications than healthy individuals. The results can be interpreted as the higher AHI, the more carotid artery calcification occurs. As these lesions can be a precursor of future strokes, 3D MDCT/CBCT images should evaluate meticulously not only extracranial but also intracranially, especially in OSA patients.

## 1. Introduction

Obstructive sleep apnea (OSA) is characterized by repetitive cycles of partial or complete upper airway collapse (hypopnea/apnea) in breathing during sleep. The obstruction generally happens in the pharynx on the posterior part of the tongue, uvula, and soft palate, or consolidation of these structures [1]. OSA leads to several obstructive episodes that cause arousal that prevents a healthy night's sleep [2]. Symptoms of OSA can divide into two sections: (1) nighttime symptoms – snoring, apnea, drowning feeling during sleep, nocturia, and inability to sleep and (2) daytime symptoms – extreme drowsiness, morning headache when you wake up, depression, irritability, memory loss, and decreased libido [3].

This sleep breathing disorder is common in the general population and remarkably frequent among patients with diagnosed cardiovascular disease. The high prevalence of OSA is mostly because of cardiovascular disease among various risk factors such as increasing age, male gender, obesity, and inactive lifestyle [4]. Due to repetitive cycles of desaturation and reoxygenation of oxyhemoglobin, an increase in blood pressure can partly explain [5]. A longitudinal population-based study known as the Wisconsin Sleep Cohort determined the prevalence of OSA to be 9% in women and 24% in men [6]. Since the Wisconsin Sleep Cohort's initiation, a dramatically related increase in the prevalence of OSA is seen over time. The Wisconsin Sleep Cohort determines the US population using data from the National Health and Nutrition Examination Survey (NHANES) adjusted for age, sex, and body mass index (BMI); the prevalence of OSA increased by 14–55% in 2007–2010 compared with 1988–1994 [7]. In 2017, Hobzova et al. [8] stated that the OSA condition has been related to depressive symptoms, and depression is frequently described as one of the daytime sequelae of OSA. They highlighted the importance of evaluating the symptoms of depression in patients with OSA.

Patients diagnosed with OSA experience stroke 3–6 times more than healthy individuals. The explanation of these strokes is undetermined, but these strokes have been variously associated with an increase in intracranial pressure and decrease in the amount of cerebral blood flow, cardiac emboli, and atherosclerosis (atheroma formation) in either the intra- or extracranial carotid artery [9]. There is a relationship between OSA and cardiovascular disease; OSA is correlated with coronary artery calcification, which is a surrogate marker for subclinical atherosclerosis [10]. In addition to coronary artery calcification, intracranial carotid artery calcification is an encouraging biomarker presenting the severity of intracranial vascular diseases [11]. In the Rotterdam study [12], internal carotid artery calcification was a huge risk factor for stroke. In addition, the intracranial carotid artery calcification reflects the overall handicap of cerebral atherosclerosis. Therefore, similar to the relationship between OSA and coronary artery calcification, intracranial carotid artery calcification might be related to OSA [13]. Harding et al. [4] have shown that patients who experienced a stroke are 8–10 times more likely to show calcified carotid artery atheromas on their panoramic radiograph than healthy individuals. Tsuda et al. reported that in obstructive sleep apnea patients, the existence of calcified carotid masses on cephalometric radiographs is indicative of cardiovascular risk [9].

Imaging techniques used in the diagnosis of OSA are diverse, including lateral cephalometric radiography, cone beam computed tomography, and magnetic resonance imaging (MRI) methods. Multidetector computed tomography has become the most preferred method for evaluating the calcification of arterial walls. Even small volumes of calcifications such as 1 mm^3^ can be seen and evaluated by cone-beam computed tomography (CBCT) [14]. Dentomaxillofacial radiologists and dentists are concerned with carotid artery calcifications since they are often observed as incidental findings on 2D panoramic and 3D CBCT scans. Thus, dentists need to diagnose these calcifications in the CBCT scans [15].  Table 1 shows the advantages and disadvantages of imaging techniques used in the diagnosis of OSA.

To the authors' knowledge, the comparison of both extra- and intracranial calcifications between OSA and non-OSA patients using CBCT has not been conducted before. Thus, we hypothesized that obstructive sleep apnea diseases may be associated with the formation of intracranial and extracranial carotid artery calcifications. Therefore, this study aimed to evaluate the prevalence and compare the intra- and extracranial carotid artery calcifications between OSA patients and healthy individuals who served as a control group.

## 2. Materials and Methods

The study was approved by Ethical Review Board (IRB-/2020/79-1100) of the Faculty of Medicine, Near East University. All procedures performed in studies involving human participants were under the ethical standards of the institutional and/or national research committee and 1964 Helsinki declaration and its later amendments or comparable ethical standards. Age, gender, body mass index (BMI), and neck circumference were recorded for all patients.

CBCT scans of 190 patients (95 patients had OSA and 95 non-OSA individuals/control group) were retrospectively collected and evaluated as well as with the body mass index (BMI) and polysomnography reports of OSA individuals at the Near East University Faculty of Dentistry and Near East University Hospital Sleep Center at the Department of Allergy, Sleep and Respiratory Diseases. The mean age for OSA individuals was 54.9 years (range: 32–84 years) and for non-OSA individuals was 45.1 years (range: 32–78 years). A total of 69 of the OSA patients were male, while this number was 53 in the non-OSA patients. In terms of systemic diseases in OSA patients, 71.5% of them were diagnosed with cardiovascular diseases; 44.8% were smokers; and 25.2% were diagnosed with diabetes. On the other hand in non-OSA patients, these percentages were 24.2%, 6.3%, and 25.3%, respectively. Patients with the confirmation of bone diseases such as osteoporosis, any bone changes regarding medicines, any skeletal anomalies, and low-quality images such as a metal artifact, motion, or inadequate bony borders were excluded from the research.

### 2.1. Image Evaluation

CBCT scans were obtained using NewTom 3G (Quantitative Radiology s.r.l., Verona, Italy). All CBCT scans were obtained in a supine position according to the strict, standardized scanning protocol used in our clinic. Patients were placed in a horizontal position; checked to ensure that their mouths were closed in a normal, natural occlusive position; and instructed to lie still throughout the length of the scan. Images were obtained using a 12-inch field of view (to ensure inclusion of the entire facial anatomy), 0.3 mm-thick axial slices, and isotropic voxels. All images were reconstructed on a 21.3-inch flat-panel color active matrix TFT medical display (Nio Color 3MP, Barco, France) with a resolution of 76 Hz 0.2115 mm pitch 10 bit. The examiners were also permitted to use enhancements and orientation tools such as magnification, brightness, and contrast to improve the visualization of the landmarks.

Cone-beam computed tomography data were evaluated by two observers (M.F. and S.A.) twice with an intermission of two weeks to determine if there is any inter- and intraobserver variability. To determine the calcifications precisely, consecutive three slices were observed using the machine's software (NNT viewer 4.2, QE Verona, Italy).

For OSA patients, CBCTs have been obtained to evaluate the pharyngeal airway. CBCTs of non-OSA individuals, which served as a control group in this study, were taken previously for various purposes such as implant surgeries, orthodontics, or paranasal sinus examinations. Control patients had no dyspnea, cough, snore, witnessed apnea, fatigue, insomnia and daytime sleepiness, and so on. There were no signs and symptoms to consider those patients as sleep apnea patients.

The evaluation of the OSA was done by a standardized program at the Department of Allergy, Sleep and Respiratory Diseases, Near East University Hospital, which consists of blood tests, blood pressure, anthropometric analysis, a questionnaire, analysis of lung function, and electrocardiogram. Evaluation of the OSA continues with ear, nose, and throat (ENT) and dentistry examination. CBCT imaging was performed at the dentistry faculty. Furthermore, overnight polysomnography, which contains an assessment of oral and nasal discharge thoracoabdominal changes and snoring; electroencephalography and electromyography of the tibia and submental; electrocardiography; oximetry; and electrooculography, was performed [16]. Calibration was performed for all patients at least two times and repeated during the night, if necessary, according to standard care procedures.

Grass Technologies (Natus Neurology Incorporated, Middleton, WI) software was used for staging and sleep recording. The apnea-hypopnea index (AHI) was characterized as the mean number of all respiratory episodes (apnea and hypopnea) per hour of sleep. The polysomnography was performed and staged by the same physician (F.R.). AHI was used for the categorization of the patients; AHI less than 5 classified as minimal sleep apnea, 5–15 mild sleep apnea, 15–30 moderate, and more than 30 indicates severe sleep apnea.

**Figure 1:**
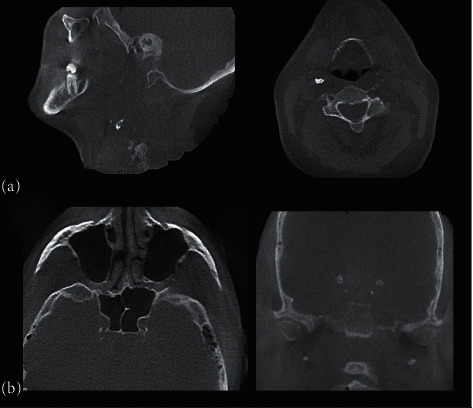
(a) Sagittal and axial CBCT images showing extracranial and (b) axial and coronal CBCT sections showing intracranial carotid artery calcifications.

Intra- and extracranial carotid artery calcifications were evaluated as unilateral or bilateral calcifications, and the grade of calcifications was noted according to a modified grading scale prepared by Erbay et al. [14]: 0 = no calcification 1 = very tiny/single dot-shaped calcifications, 2 = tiny, scattered calcifications, 3 = either thick interrupted calcifications or thin confluent calcifications, and 4 = thick contiguous calcifications. All of the CBCT images were examined in axial, coronal, and sagittal sections (Figure 1).

### 2.2. Statistical Methods and Examiner Reliability

Statistical analyses were performed using the SPSS 17.0.1 software (SPSS Inc., Chicago, IL, USA). Intra- and interexaminer validation measures were conducted. To evaluate intra-observer reliability, the Wilcoxon matched-pairs signed-rank test was performed for repeat measurements. Interobserver reliability was demonstrated by the intraclass correlation coefficient and the coefficient of variation (CV ¼ (standard deviation/mean) 100%). Values for the intraclass correlation coefficient are classified from 0 to 1. Intraclass correlation coefficient values were greater than 0.75 express great reliability, and the low coefficient of variation indicated the precision error as an indicator of reproducibility [15]. Differences in variables were evaluated using chi-square test and paired *t*-test. Differences were considered significant when *p* < 0.05. To test the hypothesis that the calcifications are associated with airway collapse in patients with sleep apnea, multiple linear regression (entry method) was used to assess the associations between calcifications and the AHI, with adjustment for gender, BMI, and neck circumference, and age.

## 3. Results

In OSA patients, 22 patients had AHI lower than 5; 20 patients had a score between 5 and 15; 19 patients between 15 and 30; and 34 patients had AHI higher than 30. For OSA patients, the mean BMI was 33.9, and the mean AHI was 27.1 (Table 2).

A total of 56.8% of the OSA patients showed at least one carotid artery calcification, whereas 13.8% of control group patients showed at least one carotid artery calcification (*p* < 0.05). The details of the comparison between extra and intracranial carotid artery calcifications between OSA and the control group are shown in Table 3. There was a significant difference between OSA patients and the control group in terms of existing calcifications (*p* < 0.05). In terms of grading of calcifications, at the right and left intracranial carotid artery, grade 1 was the most common grade of calcification in OSA patients when compared with the control group. Also, at the intracranial carotid artery, there was no grade 3 and 4 calcification found in the control group. For extracranial carotid artery calcifications, the incidence of grade 2 calcifications was higher in OSA patients.

The statistical analysis of carotid artery calcifications among OSA patients according to AHI groups is shown in Table 4. The results showed when AHI increased, the higher number of intracranial and extracranial calcifications also increased gradually. But we found a significant difference only in intracranial carotid artery calcifications when compared with the AHI groups (*p* < 0.05).

In the intracranial carotid artery (left and right), the most found calcification grade in patients with AHI < 5, 5–15, and >30 was grade 1. Patients with AHI 15–30 showed grade 2 calcification at the highest rates. At the extracranial carotid artery (left and right), there was no significant difference between calcification grades.

Moreover, intracranial artery calcifications were found bilaterally in the AHI < 5 and AHI 5–15 groups, 13.6% and 21.1%, respectively. In AHI 5–15, 15–30, and <30 groups, unilateral calcifications were observed in 25%, 21.1%, and 50%, respectively. The unilateral calcifications in the AHI groups are higher than in the control group, and the incidence of bilateral and unilateral calcification in the AHI 15–30 group was equally determined (21.1%). In terms of extracranial calcifications, patients with unilateral calcification were more common in AHI < 5 (22.7%), 5–15 (25%), and AHI > 30 (23.5%), while patients with unilateral and bilateral calcification in the AHI 15–30 group were equal (15.8%). Besides, the results also showed that the presence of at least one carotid artery calcification was higher in AHI < 30 group. These correlations were tested with multiple linear regression on AHI and calcifications, As AHI increases, the incidence of at least one carotid artery calcification increases (*p* < 0.05). BMI was also related to AHI; on the contrary, none of the other variables in the equation reached formal significance (Table 5).

## 4. Discussion

Atheromas probably occur with merged effects of hypertension and hypoxia. Hypoapneic/apneic events are associated with periodic adjustments of heart rate and blood pressure, including oxygen tension, with intense hypoxia, again and again by reoxygenation; hypoxic conditions may occur during 50% of night sleep. It is obvious that human epidemiology indicates that OSA is related to an increased risk of vascular diseases, such as stroke and acute myocardial infarction [16, 17]. Their presence and location discriminated from other similar formations such as anatomic formations, for example; epiglottis, stylohyoid and stylomandibular ligaments, and pathologic formations such as calcified lymph nodes and sialoliths. Besides, CBCT in a larger field of view raises the possibility of exploring intracranial and extracranial calcifications as incidental findings at important anatomic locations beyond the aim of the imaging process [18].

Harding et al. [4] compared the carotid atheromas with OSA patients and normal subjects on panoramic radiography, and they found that 22% of the OSA patients showed atheromas on panoramic radiography. Tsuda et al. [10] evaluated the prevalence of calcification in cephalometric radiographs of OSA patients they found that 10.4% of the OSA patients had calcifications. Harding et al. [4] and Tsuda et al. [10] found the calcifications in OSA patients less than our findings; they used two-dimensional images for evaluation. We used three-dimensional cone-beam computed tomography for examination. On a panoramic and cephalometric radiograph, carotid artery calcifications can be confused with other calcifications such as lymph node calcifications or sialoliths because, in two-dimensional radiography, it cannot be sure definitely if the location is right. Another handicap for two-dimensional radiography is artifacts. They can be easily confused with calcifications.

Koh et al. [9] evaluated the relationship between OSA severity and carotid arterial calcification by quantitative analysis using airway multidetector computed tomography. They observed that 16.6% of the OSA patients had carotid arterial calcification. Koh et al. [9] found low calcification than our findings because they used multidetector computed tomography that is three-dimensional, but the thickness of the slices is much smaller in CBCT. So small calcifications can easily be missed on multidetector computed tomography.

Damaskas et al. [19] evaluated the association between extra- and intracranial calcifications of the internal carotid artery. A total CBCT data of 705 non-OSA patients were examined. They have recorded 799 calcifications: 480 (60.1%) were intracranial calcifications, and 319 (39.9%) were extracranial calcifications. Since we evaluated 94 healthy individuals, we found 15 calcification in non-OSA patients. Six (40%) of them were intracranial calcifications, and 9 (60%) of them were extracranial calcifications. It is believed that this difference caused by their study findings is due to the inclusion of OSA patients in the general population in their studies. In their study, the observers implied that patients did not know the potential medical and dental anamnesis of the patients, so in their opinion, the presence of OSA patients in the study groups did not affect the final results. However, our study findings showed that there were significant differences between OSA patients and healthy individuals in terms of extra- and intracranial carotid artery calcification. Since systemic diseases affect the formation of calcification, the whole anamnesis of the patients must be recorded before jumping to any conclusion.

Besides, Mutalik and Tadinada [20] evaluated the relationship between extra- and intracranial carotid artery calcifications in patients without OSA using CBCT. They stated that extracranial carotid artery calcification was observed in 38% of patients, intracranial carotid artery calcification was found in 43.8%, and the prevalence of intracranial carotid artery calcifications increased in the presence of extracranial carotid artery calcifications. Woo et al. [13] studied the relationship between the OSA patients who had AHI > 30 (high-risk OSA patients) and intracranial carotid artery calcifications in patients with acute ischemic stroke using multidetector computed tomography. They used Berlin questionnaire for diagnosing OSA. They applied the Agatston method for calcium scoring. Agatston method means an area ≥1 mm and ≥2 mm continuous pixels with an MDCT density ≥130 Hounsfield units (HU) are automatically recognized as calcification by the software. Intracranial carotid artery calcification was accepted present if the Agatston score was higher than 0. They found that high-risk OSA patients showed significantly more amount of intracranial artery calcification in both intracranial arteries, symptomatic intracranial arteries, and asymptomatic intracranial arteries than the low-risk OSA patients (97.1% vs. 76.3%, *p*=0.015; 91.4% vs. 63.2%, *p*=0.005; 91.4% vs. 55.3%, *p*=0.001). Mutalik and Tadinada [20] found similar results to our outcomes.

Lee et al. [21] investigated the value of airway multidetector computed tomography in OSA patients as a prognosticator of cerebrocardiovascular disease clinically, by quantitatively analyzing carotid arterial calcification. The carotid artery calcification score was quantified with the modified Agatston method. Cerebrocardiovascular diseases occurred in 27 OSA patients out of 287 OSA patients. Patients who had cerebrocardiovascular disease showed a significantly older mean age (57.5 years vs. 54.2 years), higher prevalence of hypertension (59% vs. 34%), and carotid artery calcification (51.9% vs. 20.8%), whereas sex, other comorbid diseases, and severity of OSA were not significantly different from the non-cerebrocardiovascular disease patients. Lee et al. [22] also studied the association of carotid artery calcification on panoramic radiographs and evaluate carotid artery atherosclerosis with ultrasonography. They used panoramic radiographs for the existence of carotid artery calcifications. They measured common carotid artery intima-media thickness, carotid bulb intima-media thickness, carotid plaques, and the diameter of the common carotid artery using ultrasonography. In conclusion, they stated that carotid artery calcification on panoramic radiographs was correlated with intima-media thickness and plaque in men and positively correlated with carotid diameter in both genders. Carotid calcifications on panoramic radiographs were positively correlated with carotid atherosclerosis.

We found that as AHI increases, patients have more likely to have at least one carotid artery calcification. As AHI increases, it leads to repetitive cycles of desaturation and an increase in blood pressure. Due to this, it is more likely to have more long cycles of desaturation, collapse in breathing, high blood pressure, and more likely experience a stroke. Since these findings lead to atherosclerosis, which is correlated with arterial calcifications, it is natural to see at least one carotid artery calcification when AHI increases. Kim et al. [23] evaluated the association between objectively measured OSA severity and the presence of subclinical systemic atherosclerosis using non-invasive measurements, and they found results that support our finding of AHI and calcification. They found moderate to severe OSA were 1.6 times more likely to have ascending thoracic aorta calcification than non-OSA patients. They stated that the severity of OSA was independently associated with subclinical systemic atherosclerosis.

Other imaging modalities such as vascular ultrasound are a more convenient and direct way to evaluate atherosclerosis of extracranial carotid arteries rather than CBCT, but Friedlander et al. [17] stated that carotid artery ultrasound is tremendously accurate in detecting the presence of calcified atherosclerotic lesions of cone-beam CT volume of more than 8 mm^3^. However, it was reported that it is less accurate in detecting smaller volume calcified plaques, with relatively high false negativity.

It should be stated that none of these studies evaluated the degree of artificial intelligence concerning both intra- and extracranial calcification. Our results indicated that the incidence of these calcifications gradually increases as AHI increases that can be interpreted as OSA patients can be potential candidates for acute ischemic stroke and these patients should be followed up intensively.

Since the degree of vascular calcifications is not strongly correlated with the degree of atherosclerosis it can be stated as a limitation of our study. Another limitation of this study can be the sample size that is not large; therefore, further studies in this line of research with large samples are recommended.

## 5. Conclusion

In conclusion, OSA patients showed a higher prevalence of calcified carotid artery calcifications than healthy individuals. The results can be interpreted as the higher AHI, the more carotid artery calcification occurs. As these lesions can be a precursor of future strokes, 3D MDCT/CBCT images should evaluate meticulously not only extracranial but also intracranially, especially in OSA patients.

## Figures and Tables

**Table 1:** Advantages and disadvantages of imaging techniques used in the diagnosis of OSA.

	CBCT	MDCT	MRI	US
Advantages	Short scanning time	Short scanning time	Higher soft tissue resolution than MDCT	Radiation-free
	Lower dose than MDCT	Soft tissue assessment possible	3D evaluation of structures with no radiation exposure	Sensitive for detecting dynamic changes in the pharyngeal airspace
	Multiplanar views and 3D reconstruction	Multiplanar views and 3D reconstruction	Dynamic imaging possible	
	Providing accurate cross-sectional information	Providing accurate cross-sectional information		
	Ability to scan the entire airway, providing accurate measurement of the airway	Ability to scan the entire airway, providing accurate measurement of the airway		

Disadvantages	Amalgam and metallic restorations causing artifacts	Amalgam and metallic restorations causing artifacts	Artifacts generated by metallic hardware	Bone blocks US waves
	Higher doses than two-dimensional imaging	High dose	Longer to acquire than MDCT	The quality of results and use of equipment depending on the skills of the operator
	Inability to accurately represent the internal structure of soft tissues	Inability to image the entire pharyngeal airway in a single plane	Patient discomfort	Less spatial resolution compared to MDCT and MRI scan

**Table 2:** Distribution of OSA patients according to AHI groups.

AHI group										Mean AHI
<5		5–15		15–30		>30		Total		
*n*	(%)	*n*	(%)	*n*	(%)	*n*	(%)	*n*	(%)	
22	23.2	20	21	19	20	34	35.8	95	100.0	27.1/hr

**Table 3:** Comparison of extra and intracranial carotid artery calcifications between OSA and control group.

		Group						Chi-square analysis	
		Control		OSA		Total			
		*n*	(%)	*n*	(%)	*n*	(%)	Chi-square	*p*
Right intracranial	No calcification	88	**93.6**	62	**65.3**	150	79.4	—	**0.0001**
	1	0	0	17	17.9	17	9		
	2	6	6.4	8	8.4	14	7.4		
	3	0	0	7	7.4	7	3.7		
	4	0	0	1	1.1	1	0.5		
	Total patients	94	100	95	100	189	100		
Left intracranial	No calcification	89	**94.7**	69	**72.6**	158	83.6	—	**0.0001**
	1	1	1.1	16	16.8	17	9		
	2	4	4.3	4	4.2	8	4.2		
	3	0	0	5	5.3	5	2.6		
	4	0	0	1	1.1	1	0.5		
	Total patients	94	100	95	100	189	100		
Unilateral-bilateral intracranial	No calcification	88	93.6	52	54.7	140	74.1	38.1	**0.001**
	Unilateral	2	**2.1**	28	**29.5**	30	15.9		
	Bilateral	4	4.3	15	15.8	19	10.1		
	Total patients	94	100	95	100	189	100		
Right extracranial	No calcification	86	**91.5**	77	**81.1**	163	86.2	—	0.178
	1	3	3.2	3	3.2	6	3.2		
	2	4	4.3	9	9.5	13	6.9		
	3	1	1.1	4	4.2	5	2.6		
	4	0	0	2	2.1	2	1.1		
	Total patients	94	100	95	100	189	100		
Left extracranial	No calcification	90	**95.7**	78	**82.1**	168	88.9	—	**0.034**
	1	1	1.1	3	3.2	4	2.1		
	2	2	2.1	10	10.5	12	6.3		
	3	1	1.1	3	3.2	4	2.1		
	4	0	0	1	1.1	1	0.5		
	Total patients	94	100	95	100	189	100		
Unilateral-bilateral extracranial	No calcification	84	90.3	67	70.5	151	80.3	11.8	**0.003**
	Unilateral	6	**6.5**	21	**22.1**	27	14.4		
	Bilateral	3	3.2	7	7.4	10	5.3		
	Total patients	93	100	95	100	188	100		
At least one carotid artery calcification	No calcification	81	86.2	41	43.2	122	64.6	38.2	**0.0001**
	Calcification	13	**13.8**	54	**56.8**	67	35.4		

Values in boldface shows statistical significance (*p* < 0.05).

**Table 4:** Statistical analysis of carotid artery calcifications among OSA patients according to AHI groups.

		AHI Group										Chi-square analysis
		<5		5–15		15–30		>30		Total		*p* value
		*n*	(%)	*n*	(%)	*n*	(%)	*n*	(%)	*n*	(%)	
Right intracranial	No calcification	17	77.3	16	80.0	12	63.2	17	50.0	62	65.3	**0.045**
	1	3	13.6	4	20.0	0	0.0	10	**29.4**	17	17.9	
	2	1	4.5	0	0.0	3	15.8	4	11.8	8	8.4	
	3	0	0.0	1	4.5	3	15.8	3	8.8	7	7.4	
	4	0	0.0	0	0.0	1	5.3	0	0.0	1	1.1	
	Total calcification	4	100.0	5	100.0	7	100.0	17	100.0	95	100.0	
Left intracranial	No calcification	19	86.4	17	85.0	14	73.7	19	55.9	69	72.6	0.095
	1	2	9.1	3	15.0	1	5.3	10	29.4	16	16.8	
	2	0	0.0	0	0.0	2	10.5	2	5.9	4	4.2	
	3	1	4.5	0	0.0	1	5.3	3	8.8	5	5.3	
	4	0	0.0	0	0.0	1	5.3	0	0.0	1	1.1	
	Total calcification	3	100.0	3	100.0	5	100.0	12	100.0	95	100.0	
Unilateral-bilateral intracranial	No calcification	17	77.3	14	70.0	11	57.9	10	29.4	52	54.7	**0.005**
	Unilateral	2	9.1	5	25.0	4	21.1	17	**50.0**	28	29.5	
	Bilateral	3	13.6	1	5.0	4	21.1	7	**20.6**	15	15.8	
	Total calcification	5	100.0	6	100.0	8	100.0	24	100.0	95	100.0	
Right extracranial	No calcification	19	86.4	17	85.0	14	73.7	27	79.4	77	81.1	0.167
	1	0	0.0	0	0.0	1	5.3	2	5.9	3	3.2	
	2	2	9.1	3	15.0	0	0.0	4	11.8	9	9.5	
	3	1	4.5	0	0.0	2	10.5	1	2.9	4	4.2	
	4	0	0.0	0	0.0	2	10.5	0	0.0	2	2.1	
	Total calcification	3	100.0	3	100.0	5	100.0	7	100.0	95	100.0	
Left extracranial	No calcification	18	81.8	15	75.0	15	78.9	30	88.2	78	82.1	0.737
	1	0	0.0	1	5.0	1	5.3	1	2.9	3	3.2	
	2	4	18.2	2	5.9	1	5.3	3	15.0	10	10.5	
	3	0	0.0	1	5.0	1	5.3	1	2.9	3	3.2	
	4	0	0.0	0	0.0	1	5.3	0	0.0	1	1.1	
	Total calcification	4	100.0	4	100.0	4	100.0	5	100.0	95	100.0	
Unilateral-bilateral extracranial	No calcification	16	72.7	14	70.0	13	68.4	24	70.6	67	70.5	0.861
	Unilateral	5	22.7	5	25.0	3	15.8	8	23.5	21	22.1	
	Bilateral	1	4.5	1	5.0	3	15.8	2	5.9	7	7.4	
	Total calcification	6	100.0	6	100.0	6	100.0	10	100.0	95	100.0	
At least one carotid artery calcification	No calcification	14	**63.6**	10	**50.0**	9	52.6	7	20.6	41	43.2	**0.011**
	Calcification	**8**	**36.4**	**10**	**50.0**	**10**	**47.4**	**27**	**79.4**	**54**	**56.8**	

Bold values show statistical significance (*p* < 0.05).

**Table 5:** Multiple linear regression on the apnea-hypopnea index (AHI) coefficients and their 95% confidence intervals (95% CI) was used to assess the associations to the calcifications with adjustment for sex, body mass index (BMI) neck circumference, and age in OSA patients.

		AHI
Total calcifications	Coefficient	−2.992
	CI 95	−5.720
	*p*	0.042^*∗*^
Extracranial calcifications	Coefficient	−4.119
	CI 95	−8.063
	*p*	0.021^*∗*^
Intracranial calcifications	Coefficient	−3.444
	CI 95	−8.024
	*p*	0.037^*∗*^
Age (years)	Coefficient	0.330
	CI 95	−0.637
	*p*	0.535
Neck circumference (cm)	Coefficient	1.422
	CI 95	−1.609
	*p*	0.403
Sex	Coefficient	−9.888
	CI 95	−28.165
	*p*	0.331
Body mass index (BMI; kgxm^−2^)	Coefficient	−0.2615
	CI 95	−5.083
	*p*	0.012^*∗*^
Cardiovascular diseases	Coefficient	−4.158
	CI 95	−6.228
	*p*	0.013^*∗*^
Smoking	Coefficient	−12.009
	CI 95	−6.549
	*p*	0.570^*∗*^
Diabetes	Coefficient	−3.444
	CI95	−8.024
	*p*	0.052^*∗*^

^*∗*^shows statistical significance (*p* < 0.05).

## Data Availability

The data sets used and/or analyzed during the current study are available from the corresponding author on reasonable request.
